# Actomyosin contractility and collective migration: may the force be with you

**DOI:** 10.1016/j.ceb.2017.06.006

**Published:** 2017-10

**Authors:** Pahini Pandya, Jose L Orgaz, Victoria Sanz-Moreno

**Affiliations:** Tumour Plasticity Team, Randall Division of Cell and Molecular Biophysics, New Hunt's House, Guy's Campus, King's College London, London SE1 1UL, UK

## Abstract

Collective cell migration is essential during physiological processes such as development or wound healing and in pathological conditions such as cancer dissemination. Cells migrating within multicellular tissues experiment different forces which play an intricate role during tissue formation, development and maintenance. How cells are able to respond to these forces depends largely on how they interact with the extracellular matrix. In this review, we focus on mechanics and mechanotransduction in collective migration. In particular, we discuss current knowledge on how cells integrate mechanical signals during collective migration and we highlight actomyosin contractility as a central hub coordinating mechanosensing and mechanotransduction responses.

## Highlights

• Collective migration relies on the ability of a multicellular co-ordinated unit to efficiently respond to physical changes in their surrounding matrix.
• Conversely, migrating cohorts physically alter their microenvironment using mechanical forces.
• During collective migration, actomyosin contractility acts as a central hub coordinating mechanosensing and mechanotransduction responses.

**Current Opinion in Cell Biology** 2017, **48**:87–96

This review comes from a themed issue on **Cell Dynamics**

Edited by **Eugenia Piddini** and **Helen McNeill**

For a complete overview see the Issue and the Editorial

Available online 15th July 2017

**http://dx.doi.org/10.1016/j.ceb.2017.06.006**

0955-0674/© 2017 The Authors. Published by Elsevier Ltd. This is an open access article under the CC BY license (http://creativecommons.org/licenses/by/4.0/).

## Introduction

Cells can migrate individually or collectively as multicellular groups (reviewed in [1]). Collective migration is observed within compact and cohesive cell groups with two or more neighbouring cells that are able to migrate facilitated by long-lived cell-cell junctions [2]. Coordinated collective migration is required for the formation of tissues and organs during development of multicellular organisms. Collective cell migration is also important during adult stage for wound healing, tissue renewal and angiogenesis. Furthermore, abnormal collective migration has been linked to tumour spread.

Some principles governing individual cell migration can be applied to collective migration, even if the regulation is far more complex. Individual migration is tightly coordinated and involves actin polymerization which drives the formation of protrusive membrane structures such as actin-rich protrusions, pseudopodia, invadopodia and blebs. F-actin polymers serve as scaffold for myosin II motors and a prerequisite for actomyosin contractile activity. Activation of Rho-associated protein kinase (ROCK) downstream of Rho GTPase (Ras homolog family member A) results in activating phosphorylation of the regulatory light chain of myosin II (MLC2) [3] and inactivation of myosin phosphatase target subunit-1 (MYPT1) [4]. Phosphorylated myosin II promotes contraction of actin fibres, generating forces that enable cells to be displaced [1, 5].

On the other hand, directional polarity involving a leading edge at the front and a lagging edge at the back is needed for efficient migration. Protrusion and adhesion of the leading edge and retraction of the rear edge drive movement in the direction of locomotion [6]. Differential regulation and organization of the actomyosin machinery results in adoption of different migratory strategies, depending on cell type, cell number and tissue structure. During individual migration, high levels of adhesion at the front coupled to Rho-ROCK driven actomyosin contractility at the rear drives elongated-mesenchymal migration while elevated levels of Rho-ROCK signalling, high actomyosin contractility and low degree of adhesion result in rounded-amoeboid migration. Stimuli which alter the balance between activity and organization of actomyosin machinery, cell matrix and cell-cell adhesions results in cells switching between adhesion dependent elongated-mesenchymal modes, bleb based rounded-amoeboid modes and collective modes [1, 7, 8, 9]. This “plasticity” is particularly relevant in the context of cancer cells, as it offers cells the ability to move in diverse extracellular environments [1, 2].

In contrast, during collective migration cells migrate as cohesive groups involving direct cell-cell contacts, as seen in epithelial cell sheets; or as multicellular streams with transient cell-cell contacts, as observed during neural crest cell migration [1, 2, 10, 11]. Branching morphogenesis in the mammary gland, vascular sprouting and border cell migration in Drosophila [12] are all physiological processes that require coordinated collective cell migration. In pathological processes such as cancer, tumour cells can move using multicellular streaming, tumour budding and collective invasion [1, 13].

During collective migration multiple cells migrate in the same direction at a similar speed behaving as one co-ordinated unit [1, 2, 14]. The direction and speed are determined by one or several leader cells with mesenchymal characteristics. The basic principles of front-to-rear polarity during single-cell migration can also be applied to collective movement where the leader cells extend actomyosin-mediated protrusions to generate integrin-based forward traction [15]; proteolytically degrade the surrounding tissue structure [16, 17] and re-align the extracellular matrix (ECM) to guide the group [18, 19]. Following cells are passively dragged behind along the established migration track by cell-cell adhesion [20, 21], reinforcing the ECM alignment [22]. The migratory group behaves as one “supra-cellular unit”, in which cytoskeletal protrusion and actomyosin contractility are mechanically linked through cell-cell junctions and span across several cells [15, 21, 23, 24]. The co-ordinated response and migration of these cells relies on communication either through diffusible factors or by the local remodelling of the ECM.

**Figure 1:**
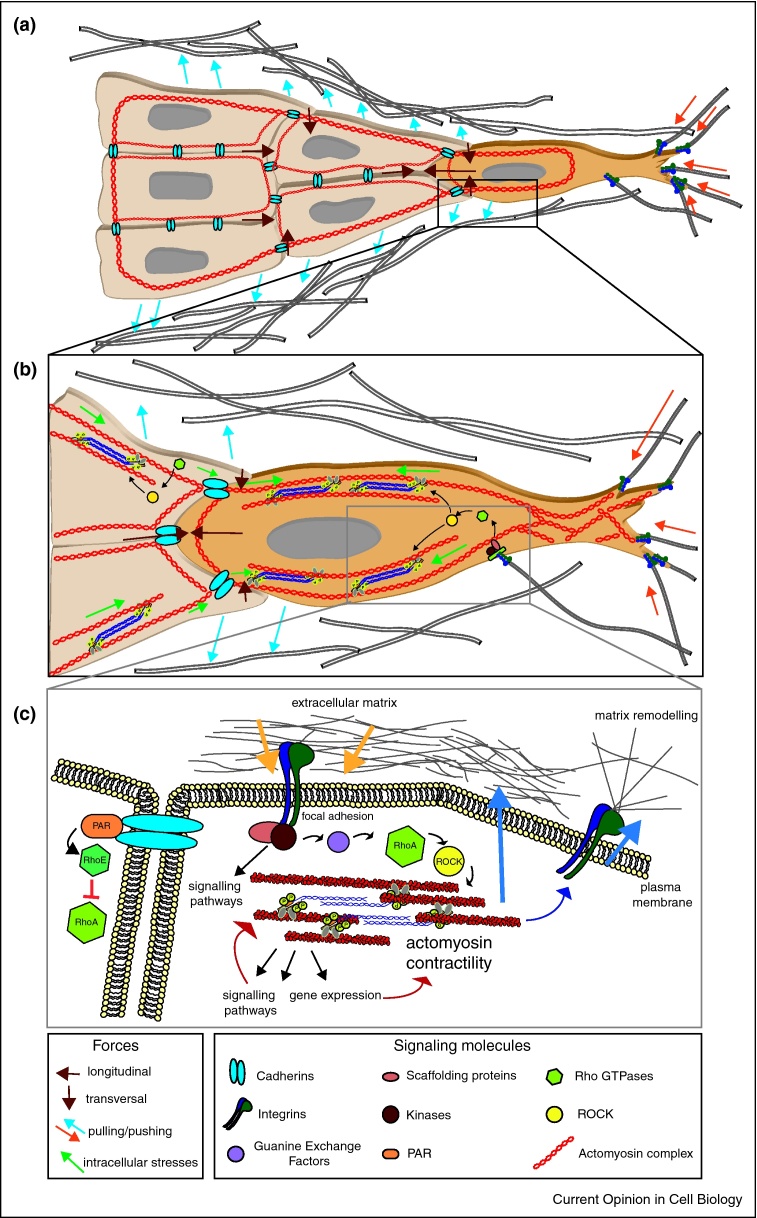
**Actomyosin contractility as a key hub in mechanosensing during collective migration.** Diagram showing the mechanical and biochemical signalling during collective cell migration. **(a)** Leader cells exert pulling forces on the matrix (red arrows) while follower cells dragged behind push away the matrix (blue arrows) to form tracks. Actomyosin cytoskeleton linked across cells facilitates the transmission of these forces from the cell to the matrix resulting in matrix alignment. **(b)** Differential organization of actin and levels of actomyosin contractility are required for polarisation and maintenance of leader cells. Cells exhibit low actomyosin contractility levels at cell-cell junctions. The variations in activity and localisation of actomyosin result in intracellular stresses (green arrows) which are crucial for matrix remodelling. **(c)** Cells not only sense physical changes in the matrix (yellow arrows) but also drive changes that result in matrix remodelling in turn (dark blue arrows). Rho-ROCK signalling downstream of integrins is the central player in the reciprocal relation between the cell and their matrix.

Mechanosensing and mechanotransduction are the processes by which cells sense changes in the physical environment and translate those mechanical stimuli into biochemical signals [25] (Figure 1, Table 1). Cells migrating within multicellular structures are subjected to different forces including tensile forces, compressive forces, hydrostatic pressure and fluid shear stress. On the other hand, collectively migrating cells apply traction forces to the extracellular environment. In this Review, we focus on the mechanics and mechanotransduction of collective cell migration and discuss how actomyosin contractility could be considered a central hub coordinating mechanosensing and mechanotransduction responses during such collective migratory processes. For a review on the developmental role and *in vivo* regulation of collective cell migration and other cell re-arrangements, see the accompanying Review from R Fernandez-Gonzalez.

**Table 1:** Summary of signalling pathways controlling collective migration

Type	Protein	References
Adhesion receptors	Integrin β1	[15, 34, 35, 36••, 100]
	DDR1	[23]
Cadherins	E-Cadherin	[57^••^]
	P-Cadherin	[58^••^]
Catenins	p120 catenin	[57••, 51]
	α-Catenin	
	β-Catenin	
Tyrosine kinase signalling	FAK	[41, 42]
	Src	[64]
RhoGEFs	SCRIB	[37]
	βPIX	[58^••^]
	Par3/Par6	[23]
	TIAM1	[39]
Rho GTPases	Rac	[36••, 96, 101]
	Cdc42	[37]
	RhoA	[63, 44]
	RhoE	[23]
RhoGTPase effectors	ROCK	[97]

## Actomyosin contractility, adhesion and mechanotransduction in leader cells

Establishment of cell adhesions and ECM imposed strains promote cytoskeletal re-arrangements, leading to signalling cascade activation and gene expression changes [26, 27, 28, 29]. Sensing of the environment is facilitated by the combined action of integrins- and actomyosin contractility. Cell adhesion molecules sense localised changes in substrate and this is crucial for translation of cytoskeletal forces into motion. Meanwhile, actomyosin cables spanning across the length of the cell allow for the determination of overall stiffness of the ECM [30] (Figure 1a, c).

Most of the migratory surfaces including collagens, laminins and fibronectin can engage cellular integrins [31]. Integrin-mediated adhesions significantly influence collective cell migration [32, 33]. In particular, β1-integrins have been implicated in collective movement of endothelial cells, astrocytes and epithelial cells [15, 34, 35, 36••]. β1-integrin recruits and activates small Rho GTPases Rac (Ras-related C3 botulinum toxin substrate 1) [36^••^] and Cdc42 (cell division cycle 42) through GTPase activators guanosine exchange factors (GEFs) -such as SCRIB (Scribble) and βPIX (PAK-interacting exchange factor beta/ARHGEF7) or Par3 (partitioning defective 3), and TIAM1 (T-cell lymphoma invasion and metastasis 1) [37, 38, 39, 40]; as well as intracellular kinases such as FAK (focal adhesion kinase, also known as PTK2) and Src within the leading cells [41, 42]. Differential Rho GTPase activation in leader cells regulates actin polymerization, actomyosin contractility and force transmission [43]. Spatio-temporal mapping of Rho GTPase activity at the leading edge suggested that upon RhoA engagement, the collective would move through a ‘leading-edge pulling’ mechanism where the leader cells pulls/drags the peripheral structure. However, disabling RhoA would lead to a “forward pushing” mechanism from behind (follower cells pushing) [44, 45]. Conversely, the rear of the leader cell retains cell–cell junctions and a delicate balance of RhoGTPase activity and actomyosin levels is established [23, 43, 44, 46••, 47].

Cadherin-mediated adhesions exhibit an antagonistic relationship with integrin-based adhesions and offer polarisation and directional cues during collective motility [48, 49, 50, 51]. Anisotropic distribution of cadherin-mediated cell-cell junctions is sufficient to induce cellular polarisation by promoting cell contractility at the rear end of the cell. Cadherins also suppress protrusion formation and the adherens-junction mediated constitutive engagement of integrins with ECM [48, 52, 53, 54, 55]. This is reinforced by transversal RhoA-mediated contractility which is responsible for confining multicellular strands and restricting *de novo* leader cell development to the lateral margins [23, 56].

Cadherins at the mechanosensitive adherens junctions are crucial for maintaining the integrity of migrating cellular sheets by interacting with and controlling the actomyosin network via catenins (p120-, α- and β-catenin) [51, 57••]. The balance between different cadherins is responsible for different behaviours of collectively migrating cells. In particular, pulling forces reinforce E-cadherin-mediated junctions [57^••^], while P-cadherin is responsible for inducing polarisation and collective migration via an increase in the anisotropy and strength of mechanical forces [58^••^]. Mechanical regulation by P-cadherin is mediated by P-cadherin/β-PIX/Cdc42 axis and is absent in cells expressing E- or R-cadherin. Force anisotropy induced by P-cadherin increases both intercellular stress and traction forces [58^••^].

Intracellular pulling forces of the leading cells result in the re-localisation of the tumour-suppressor protein merlin from cell-cell contacts to the cytoplasm, leading to polarised Rac1 activation and lamellipodium formation [46]. Intercellular pulling forces in follower cells propagate this merlin/Rac1-mediated lamellipodium generation at a multicellular scale [46]. On the other hand, RhoA signalling is required for generating actomyosin-mediated forces towards the substrate. These forces are crucial for facilitating collective migration by enabling longitudinal force transmission from leader to follower cells through supracellular actomyosin coupling [45].

During collective migration, cytoskeletal remodelling is sustained by changes in transcriptional programs. Maintenance of leader cell identity is dynamically regulated via Notch1-Dll4 lateral inhibition. Mechanical stress in a migrating epithelium inhibits Dll4 expression and leader cell formation [59], while Rho signalling promotes actomyosin contraction at the cell rear. Long-term polarisation of leader cells is maintained via gene expression changes involving the Hippo pathway [60]. Components of the Hippo pathway localise to contacts between border cells inside the cluster and signal through the Hippo and Warts kinases to polarise actin in border cells. Furthermore, transcriptional programs regulated by STAT3 (Signal transducer and activator of transcription 3) [61] and YAP (Yes associated protein 1) [29] within cancer-associated fibroblasts have been shown to support collective migration of cancer cells. In this case, the leaders and the followers have different cellular origins but work as a unit; that is, leaders are fibroblasts and followers are cancer cells. Interestingly, transcription factor HIF1α (Hypoxia inducible factor 1 alpha subunit) has been shown to promote a switch from collective to highly contractile cancer amoeboid mode of migration under hypoxic stimuli [9].

Therefore tight regulation of adhesion molecules, actomyosin activity and specific transcriptional programs in leader cells are crucial for efficient collective migration.

## Actomyosin contractility, adhesion and mechanotransduction in follower cells

Transmission of the large contractile forces generated by the leader cells drags the follower cells, establishing a tissue-scale coherent force profile and setting the peripheral actin cable under tension (Figure 1a). Myosin II is required to integrate and organize mechanical tension at the supra-cellular level, preventing follower cells from extending migratory protrusions and maintaining polarity [44, 45, 56].

At the sub-cellular level, follower cells are connected to the leader cells via polarised VE-cadherin-rich membrane protrusions or ‘cadherin fingers’. These extend from the rear of leader cells and are engulfed by the front of follower cells [62^••^]. RhoA activity is maximal at the edges of the fingers, indicating its involvement in the regulation of the actomyosin cables [44, 63]. This generates opposite membrane curvatures and asymmetric recruitment of curvature-sensing proteins. The region of cadherin finger engulfment is associated with VE-cadherin/catenin complexes and Arp2/3-driven actin polymerization, resulting in formation of a lamellipodia-like zone with low actomyosin contractility [62^••^].

Optimal levels of actomyosin contractility at cell-cell junctions in collectively invading cancer cells can be regulated by collagen-activated tyrosine kinase receptor DDR1 (discoidin domain receptor family, member 1). RhoE localised to cell-cell contacts by the DDR1–Par3/Par6 complex antagonizes excessive ROCK-driven actomyosin contractility and maintains collective organization [23] (Figure 1c). DDR1 has also been shown to stabilize membrane localisation of E-cadherin by inhibiting the integrin β1-Src-mediated clathrin-dependent endocytosis pathway [64]. This study showed that DDR1 knockdown activated Src activity in a β1-integrin-dependent fashion. Active Src would, in turn, phosphorylate E-cadherin on the p120-catenin binding site, leading to clathrin-mediated endocytosis of E-cadherin, followed by ubiquitination and subsequent degradation [64]. On the other hand, when cell-substrate adhesion is low, intercellular adhesions in migrating clusters are held together by actomyosin bridges and are subjected to high tension [65].

Actomyosin-mediated mechanical organization at the individual cell level will identify leader vs followers within the collective migratory group. This relationship between tension, actomyosin contractility and Rho activation is an example of common mechanisms that apply to both collective as well as individual migrating cells [44, 66].

## Cell-matrix interactions during collective migration

Migrating cells display an exceptional ability to adapt to different environmental conditions [2, 30, 67]. Continuous reciprocal communication between cells and their surrounding matrix is established as migrating cells remodel the matrix. In return, changes in matrix physical properties will determine how cells migrate. How these two phenomena are integrated and resolved over time is still not fully understood, neither for individual nor for collectively migrating cells.

## Inside-out: cells generate force to remodel the matrix

Transmission of cell traction forces to the ECM occurs via cell adhesions and results in alterations in ECM density, stiffness and architecture. Contraction of surrounding matrices results in generation of anisotropy in the pericellular matrix [68]. Mapping of the local cell-induced matrix remodelling has revealed the heterogeneous, myosin-dependent remodelling of ECM fibres surrounding the cell to facilitate directional migration [69]. Furthermore, migrating collectives, including normal epithelial cells, cancer cells, and primary organoids, have been shown to propel themselves through fibrillar matrices by cyclical actomyosin-driven pulling-relaxation cycles on impeding fibres [19]. In addition, cells in collective groups exert balanced waves of mechanical stress between cadherin-based intercellular junctions [70]. As a result, each individual cell generates force towards the substrate during collective epithelial migration [71] (Figure 1a,b).

At the multicellular cluster level, highly contractile cells exert detectable pushing, pulling and rotating forces onto the environment leading to vigorous matrix remodelling [72, 73], particularly at the leading edge. Migration of multicellular cohorts through collagenous matrices involves spatially localised, long-range dynamic pulling forces; while tensile forces increase at the invasive front of cohorts. This serves a physical, propelling role as well as a regulatory role by conditioning cells and matrix [74•, 75] and resulting in ECM alignment, creating microtracks for further migration.

The number of cells in a collective is proportional to the active tensile modulus of an epithelial sheet [76^•^], which cooperatively strains the local environment and determines tissue tension and movement. In fact, collectively migrating cells induce a strain-stiffening response of the matrix that is 4-fold higher than individually migrating cells in a β1 integrin- and actomyosin-dependent manner [74^•^]. The extent of strain stiffening at the leading edge of migration is determined by cell type, multicellular cooperativity, integrin availability, and actomyosin contractility. This strain-induced realignment and densification of ECM generates a “travelling wave” of stiffened substrate reinforcing mechanosensing and promoting self-steering of migration [74^•^].

As key receptor molecules linking the extracellular matrix to the intracellular cytoskeleton, integrins play an essential role in transmitting tension in developing tissues, since they regulate force transmission. Integrin-containing adhesive structures facilitate basal cell-ECM adhesions that provide resistance to apical cell displacements during dorsal closure *in vivo*, which generates a continuous epidermal sheet over the dorsal surface of the *Drosophila* embryo [77^•^]. The amount of basal adhesion is inversely correlated with apical force transmission while perturbing cell-ECM adhesions impairs dorsal closure.

When cells are challenged with tubular geometries, contractile forces are responsible for collagen re-alignments localised to specific regions of the tissue [75]. Cells generate narrow strips (50–100 μm) of aligned collagen that, in turn, regulate the directionality of migrating cohorts, suggesting that tissue geometries generated during collective migration may be self-referential. Interestingly, collective migration of non-neoplastic cells generates highly restricted and directional forces and matrix alignment; while forces produced by migrating cancer cohorts appear to be diffuse and delocalized [75]. While developmental and wound healing processes are very organized, that level of organization may not be possible within a developing tumour of heterogeneous and highly aberrant genetic makeup.

On the other hand, tumour spheroids have been shown to induce integrin-independent radial orientation of the surrounding collagen fibre network up to a distance of five times their radius, correlating with local tumour cell migration behaviour [78, 79]. This radial alignment is facilitated by Rho-ROCK driven actomyosin contractility and offers additional guidance cues to promote tumour cell invasion [80]. Radial matrix alignment by tumour cells also promotes and is required for directional migration of microvascular endothelial cell towards tumour cells, which further promotes angiogenesis [79].

An interesting observation is that cells migrating during wound healing responses exhibit different mechanical patterns to migratory multicellular cohorts. At early stages of the wound healing process, leading actin protrusions generate traction forces that point away from the wound, indicating that wound closure is initially driven by cell crawling [81]. However, later stages involve the cooperation of cell crawling and contraction of a supra-cellular actomyosin ring at the leading edge, since traction forces point towards the wound with strong radial and tangential force components [81]. Tension is transmitted by a heterogeneous actomyosin ring to the underlying substrate through focal adhesions [81, 82••]. While an actomyosin-dependent “purse-string” mechanism had been previously proposed as the mode of wound closure [83], recent evidence suggests that the increase in force relies on large-scale remodelling of the suspended tissue around the gap [65, 82••]. Interestingly, efficient formation and contraction of such actomyosin-based purse-strings is limited to cell types capable of wound healing (for example, embryonic epidermal cells), while epithelial cells such as MDCK are unable to efficiently close similar non-adhesive gaps [82^••^]. This specificity could arise from differences in the organization and strength of intercellular adhesion in different cell types with specific functions.

Collectively, these studies show how cell-dependent matrix remodelling offers directional cues to facilitate migration, thus generating a mechanical feedback loop.

## Outside-in: cells respond to physical changes in the ECM

Efficiency of collective migration is influenced by changes in the physical properties of the extracellular environment such as confinement, stiffness and surface geometry [30] (Figure 1a) as well as by application of external forces such as cyclic loading.

Increasing collagen density, independently of matrix stiffness, induces cells to switch from single-cell to collective invasion modes. Conversion to collective invasion includes gain of cell-to-cell junctions, supra-cellular polarisation and joint guidance along migration tracks [12, 84]. Nevertheless, matrix alignment can sustain both single cell and collective cell invasion even in absence of ECM proteolysis [85, 86]. Cells can measure overall and local stiffness of their substrate allowing them to migrate up gradients of increasing elastic modulus [30, 69]. Leader cells are more motile than follower cells on softer substrates. As a result, while migrating on softer substrates, increased tension across the migrating sheet has been measured with periods of actomyosin-dependent retraction [87]. During wound healing, wounding initiates a wave of motion coordination from the wound edge into the sheet accompanied by a polarised front-rear myosin II gradient and coupling of contractile forces between neighbouring cells [88]. The rate and range of propagation is proportional to substrate stiffness. In fact, increased collagen stiffness, via cross-linking, directly increases tumour progression, invasion and metastasis [89, 90].

Substrate geometry offers directional cues to migrating cells. During epiboly, geometric distortion of embryos results in non-uniform migration and realignment of the anterior-posterior (AP) axis towards the new long axis of the embryo [91]. Loss of actomyosin network homeostasis and contractile activity also leads to unusual cell organization and intestinal tissue defects [92^•^]. Tension generated by the contractile actomyosin ring is not only important for individual cell organization, but also for epithelial monolayer maintenance and co-ordination of epiboly on both the organismal and cellular scales [91, 92•].

Cyclic loading is the application of repeated or fluctuating stresses or strains. Migrating cells actively remodel and reorient their cytoskeleton in response to cyclic loading (Figure 1a,b). Over long time periods, cells may adapt to accommodate ECM deformations, thus, actively relaxing the stress further towards the original (pre-loading) values [93]. The ECM strains and associated stresses can induce changes in signalling cascades and gene expression via cytoskeletal remodelling [28, 94], where YAP-TAZ signalling has been shown to play a key role [26, 27, 29, 95].

As described in previous sections, cell-adhesion to the substrate determines the forces generated by the cytoskeleton to displace the cell [30]. Morphogenetic movements during development may involve migrating cells translocating within other cellular sheets [30]. In this situation, attachment to the substrate is mediated by cadherins, which are capable of mechanosensing [13, 65]. In such scenarios involving the migration of cells over a layer of immobile cells, E-cadherin-mediated adhesion between the two layers functions as a feedback loop regulating Rac and actin assembly, which stabilizes forward-directed protrusion and directionally persistent movement *in vivo* [96]. To counteract increased constriction by surrounding cells, ROCK-mediated myosin II activity increases at the cluster periphery [97]. Therefore, surrounding tissue made of matrix or of cells imposes forces that have to be balanced for collective migration to occur.

## Future perspectives

Efficient collective migration requires cells being able to exert pushing and pulling forces to remodel the ECM and at the same time, to counteract external forces, both from the ECM and neighbouring cells (Figure 1a,b,c). Actomyosin contractility lies at the core of this force-sensing and force-generating machinery, acting as a central hub (Figure 1a,c). Actomyosin needs to be coordinated at the cellular and at the multi-cellular level allowing large groups of cells to behave as a single unit. The supra-cellular coordination of cell adhesion and actomyosin contractility across individual cell boundaries is crucial for the collective to successfully migrate.

In the last few years, collective cell migration has gained increasing interest and this has allowed great progress in a field that poses more challenges than studying individual cell migration. Complex mechanisms of signal integration at the supra-cellular level are crucial during developmental processes, wound healing and tumour dissemination. Deeper understanding of the spatiotemporal mechanical regulation of collective migration is needed at the single cell level. How mechanical signals impact the nuclear compartment differently in leader and follower cells remains poorly defined. In cells migrating individually, actomyosin contractility establishes strong feedbacks with transcription factors such as STAT3 [61, 98], SMAD2 [99] or HIF1α [9] to self-sustain high actomyosin activity. It is also clear that YAP-TAZ is a major mechanosensor connected to the actomyosin machinery [26, 29]. Whether these and other transcription factors are crucial to transmit and sustain signals from leader cells to follower cells (and vice versa) in response to mechanical changes will be an interesting area of research. A combination of single cell imaging using FRET probes to monitor localised Rho GTPase activity, single cell transcriptomics and atomic force microscopy to measure forces will allow elucidation of such a complex question.

## References and recommended reading

Papers of particular interest, published within the period of review, have been highlighted as:

• of special interest
•• of outstanding interest
